# Focus gender – medical students' gender-specific perception and attitudes towards the burdens of everyday student life

**DOI:** 10.3205/zma001308

**Published:** 2020-03-16

**Authors:** Verena Steiner-Hofbauer, Mesküre Capan Melser, Anita Holzinger

**Affiliations:** 1 Medical University Vienna, Research Unit für Curriculumentwicklung, Vienna, Austria

**Keywords:** gender differences, medical students, burden, performance pressure

## Abstract

**Introduction: **The aim of this study was to investigate if female and male medical students perceive burdens differently and if students of both sexes assess their capability to stand performance pressure differently.

**Material and Methods:** In 2017, 2^nd^ (n=424, 53% female) and 6^th^ (n=161, 46.6% female) year students at the medical university of Vienna were surveyed using a fully structured questionnaire.

**Results: **In 2^nd^ year, female students felt significantly more often that they could not measure up to study requirements than male students (87,5% vs. 94,4%). Performance pressure was perceived as major problem by male (45,5%) and female (50,9%) students while in 6^th^ year the number was only half as high than in 2^nd^ (24%, 18,4%). In 6^th^ year significantly more female than male students were complaining about competition between students (33,3% vs. 8%). Half of the students shared the view that there is no difference between men and women in the capability to deal with performance pressure. Most of the other half state that men are superior to women in handling performance pressure. In both groups significantly more male than female students were convinced that they are superior to the other sex in handling performance pressure.

**Conclusion: **Perception of problems is similar in male and female students. While in objective assessments female students perform equally to male students they consider themselves less competent and are more inclined to doubt their capability.

## Zusammenfassung

*Zielsetzung:* Thema dieser Studie war, inwieweit weibliche und männliche Medizinstudierende Belastungen unterschiedlich wahrnehmen und ob die Fähigkeit beider Geschlechter mit Leistungsdruck im Studium umzugehen, von weiblichen und männlichen Studierenden unterschiedlich eingeschätzt wird.

**Methodik: **In einer Querschnittstudie der Medizinischen Universität Wien wurden 2017 424 Studierende (53% weiblich) des 2. Studienjahres, sowie 161 (46,6% weiblich) Studierende am Ende des 6. Studienjahrs mit Hilfe eines voll strukturierten Fragebogens befragt.

**Ergebnisse: **Zu Beginn des Studiums fühlten sich weibliche Studierende signifikant seltener den Anforderungen des Studiums gewachsen (87,5% vs. 94,4%), Leistungsdruck stellte das größte Problem für Studentinnen (50,9%) wie Studenten (45,8%) dar. Am Ende des Studiums klagten signifikant mehr weibliche als männliche Studierende über Konkurrenz zwischen den Kommilitoninnen bzw. Kommilitonen (33,3% vs. 8%). Die Hälfte der Studierenden war der Ansicht, dass zwischen beiden Geschlechtern keine Unterschiede im Umgang mit Leistungsdruck bestünden, in der anderen Hälfte allerdings überwog eindeutig die Meinung, dass Männer diesbezüglich Frauen überlegen seien. Zu Beginn und am Ende des Studiums waren signifikant mehr Studenten als Studentinnen davon überzeugt, persönlich besser mit Leistungsdruck umgehen zu können als Studierende des anderen Geschlechts.

**Schlussfolgerung: **Frauen und Männer erleben Belastungen weitgehend ähnlich. Obwohl weibliche Studierende die gleichen Leistungen erbringen wie ihre männlichen Kollegen, haben sie weniger Vertrauen in ihre Fähigkeiten.

## Introduction

In the 2017 winter semester, 4121 female and 3783 male students were enrolled at the Medical University of Vienna. Even though female students outnumber their male colleagues, and laws have been enacted that should be fostering female careers, the number of women is decreases during continuous medical education. At the university, this gap is especially salient. In 2017, 240 female and 298 male residents were working at the Medical University of Vienna; in 2016 37 men and only 9 women habilitated [1]. Across Austria, only 20% of all professorships were held by women in 2016 [1], [2]. The assumed reasons for this are manifold. Among other things, maternity is repeatedly mentioned as a career obstacle, but Abele [3] showed in a study on career development that even childless women were less successful than men, one and a half years after starting a career. Occupational or structural conditions, burdens such as night shifts, strict hierarchical structures, competitive thinking and ever-increasing demands on the performance of individuals lead to doubts about the choice of medicine as a profession as well as to exhaustion and frustration. In addition, personality factors such as excessive self-doubt, lack of self-confidence or an overly critical attitude towards one's own performance also contribute to professional dissatisfaction [4], [5], and in connection with gender-specific discrimination, often lead to negative career developments [6], [7], [8]. Gender stereotyped attitudes can influence the behavior and assessment of oneself and others. Kristofferson [9] showed that men are often still considered more competent and important than women by people of both sexes, and that medicine as a profession and the skills associated with it are historically and culturally linked to masculinity. It is obvious that gender-specific attitudes already matter during medical education and that the way in which female and male students deal with burdens and demands may therefore differ. International studies show that female students feel more burdened by the demands of medical studies and feel more often impaired in their mental health [10], [11], [12], [13]. Performance requirements and performance pressure such as dealing with large amounts of learning material [14], [15], competition between fellow students [14], but also social aspects such as difficulties in finding social contact, can be experienced as stressors [14], [15]. In addition, many students face financial problems [14], [15]. The feeling of having to spend too much time on education [15], [16] and therefore finding too little rest can lead to a negative work-life balance, impair mental health and result in depression [14], [16], [17]. In this study, we investigated whether female and male students perceive demands and burdens such as performance requirements, competition, social contact or financial problems differently, and whether students perceive these demands and burdens differently at the beginning of their studies in the context of medical school in contrast to at the end of their studies, after clinical training. Additionally, we investigated whether female and male students experience disadvantages because of their gender differently. Furthermore, the agreement on gender-specific statements on the abilities of women and men in dealing with performance pressure was examined in order to determine to what extent gender and stereotypical ascriptions to gender roles still influence everyday student life. We were particularly interested in the following questions:

1. **Difficulties/problems perceived by students:** Do female and male students differ in the problems they perceive in connection with their studies?
2. **Study requirements:** To what extent do female and male students feel up to the demands of their studies? Are female and male students equally satisfied with their work-life balance and are there differences in the amount of work they do?
3. **Gender discrimination:** Do female or male students feel disadvantaged in their studies because of their gender?
4. **Gender-specific handling of performance pressure:** How do students assess gender-specific statements on the ability to handle performance pressure? Are there gender differences?

## Materials and methods

In a cross-sectional study, 585 students of the Medical University of Vienna were surveyed at the beginning and at the end of their studies (2^nd^ year, end of 6^th^ year). These two dates were chosen because students in their 6^th^ year had already been able to gain insight into daily clinical work during their clinical internship (“klinisch-praktisches Jahr”), while students of the 2^nd^ year gained experience mainly within the university context. 424 students in the 2^nd^ year (group 1, 53% female), and 161 students in the 6^th^ year (group 2, 46.6% female) took part. The response rate for the 2^nd^ year was 65.9%; for the 6^th^ year, 45.9%. 

The Ethics Committee and the Data Protection Commission of the Medical University of Vienna approved the study. The survey was conducted in 2017 following a compulsory class in the 2^nd^ year (course: Medical Conversation) and after a mandatory assessment in the 6^th^ year (course: Return week). Participation in this paper pencil survey was voluntary and anonymous. The choice of compulsory courses ensured the presence of as many students as possible. The survey using a standardized questionnaire provides a broad overview of students’ opinions.

## Questionnaire

The questionnaire was self-developed by the authors. The sociodemographic data was limited to the gender, which was collected as “male” and “female”. Based on literature [14], [15], [16], [17], we defined performance requirements, financial problems, competition, problems with social contacts as problems; also “no problems” could be selected by ticking on a list. Multiple answers were possible. Study requirements were determined by the statements “I can cope well with the demands of my studies” and “I am satisfied with my work-life balance”. The statements could be rated on a four-point likert scale (0=no, absolutely not; 1=no, rather not; 2 yes, rather; 3= yes, absolutely). Additionally, we asked the question: “How many hours per week do you spend on study matters?” The following item captured the subjectively perceived disadvantage: “It seems to me that I was disadvantaged during my studies/training because of my gender”. The statements on the gender-specific handling of performance pressure were: “I think that men are generally better at handling performance pressure than women are.”; “I think that women are generally better at handling performance pressure than men are. ”; “I think that I personally am better at handling performance pressure than persons of the opposite sex are.”. Respondents were able to rate the statements on a four-point likert scale (0=no, absolutely not; 1= no, rather not; 2= yes, rather; 3= yes, absolutely).

### Statistical analysis

SPSS version 24.0.0.0 was used for data analysis. Gender differences were tested for statistical significance using the Chi-square test. For analysis the answers “no, absolutely not” and “no, rather not” as well as “yes, rather” and “yes, absolutely” were combined to a dichotomous answer set “agreement” and “rejection”. This ensures sufficient cell frequency and ensures better interpretability.

## Results

### Difficulties/problems experienced by students

**Figure 1:**
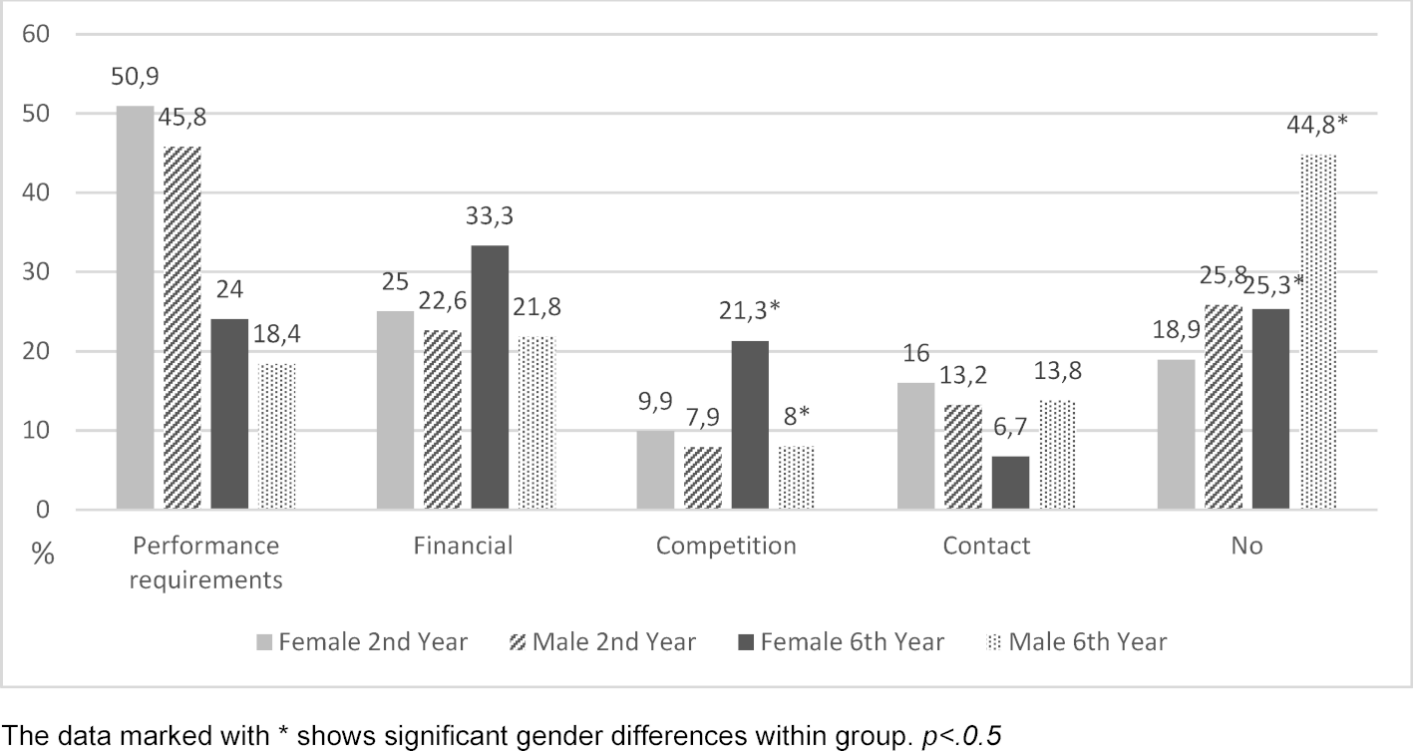
Day to day problems of female and male students of the 2^nd^ and 6^th^ year group.

The main problem second year students report is performance requirements: 50.9% of female students and 45.8% of male students state this (see figure 1 (Fig. 1)). In the sixth year, however, only about half as many students suffer from it. There are no significant differences between the sexes in either year. In second year, about a quarter of the surveyed students are affected by financial problems; in the sixth year, the number is significantly higher among female students (33.3%) than among male students, for whom the proportion remains roughly the same. While competition among 2^nd^ year students generally plays only a minor role, the problem seems to be more noticeable for women towards the end of their studies: in the 6^th^ year, more than 20% of women suffer from it, compared to only 8% of men (χ^2^(1)=5,838, p<.05). Difficulties in finding social contact with others are rare. Male students report more often that they have no problems than female students. This tendency is statistically significant in the 6th year group, where about half of male students, but only a female quarter of the students, report to have “no problems” (χ^2^(1)=6,660, p<.05) (see figure 1 (Fig. 1)).

#### Study requirements 

In the 2^nd^ year, female students feel less able to deal with the demands of their studies than male students (χ^2^(1)=5,741, p<.05) (see table 1 (Tab. 1)). In the 6^th^ year, this difference does not exist. Almost all male and female students cope well with the study requirements. There are no significant gender differences regarding satisfaction with the work-life balance, neither among students in their 2^nd^ year nor among students in their 6^th^ year. Among women, there is an insignificant tendency towards greater satisfaction among 6^th^ year female students (77% versus 66%). Female students in the 2^nd^ year spend 42.6 hours per week on their studies; significantly more hours than male students do (38.5 hours) (t(382)=-2,179, p<.05). On the other hand, male students in the 6^th^ year spend 42.8 hours per week on their studies; significantly more hours than their female colleagues (37.8 hours) (t(150)=-1,723, p<.05) (see table 1 (Tab. 1)).

#### Gender discrimination

Students in the 2^nd^ year group rarely perceive disadvantage because of gender. Only 5.6% of female and 4.1% of male students feel disadvantaged because of their gender. It is noticeable that this impression is almost three times more prevalent among both sexes in the 6^th^ year group. 15.8% of female (χ^2^(1)=7,805, p<.05) and 11.5% of male (χ^2^(1)=5,442, p<.05) students feel disadvantaged because of their gender. Both differences are statistically significant.

#### Gender stereotypes: attitudes towards dealing with performance pressure 

About half (51.7%) of the 2^nd^ year students denied both the statement that men could generally cope better with performance pressure and the statement that women were better able to do so. Therefore, about half of the students see no difference between the sexes regarding these abilities. Male students state this more often than female students, but the difference is not statistically significant (57.8% vs. 46.5%; χ^2^(1)=2.746, p>0,05). 

If a gender difference is perceived, both male and female students (38.6% and 45%, respectively) more frequently anticipate men as more competent in dealing with performance pressure. Only a minority (20.3% of women and 5.1% of men) adopt the opposite view, namely that women are superior to men in coping with performance pressure. Female students take this view significantly more often than male students do. A similar pattern can also be found among 6^th^ year students. Here, 62.3% of male and 50.6% of female students deny a gender difference in coping with performance pressure (χ^2^(1)=2.181; p>0,05). The students who perceive a gender difference generally believe that men, rather than women, can cope better with the performance pressure. This applies equally to male and female students.

**Figure 2:**
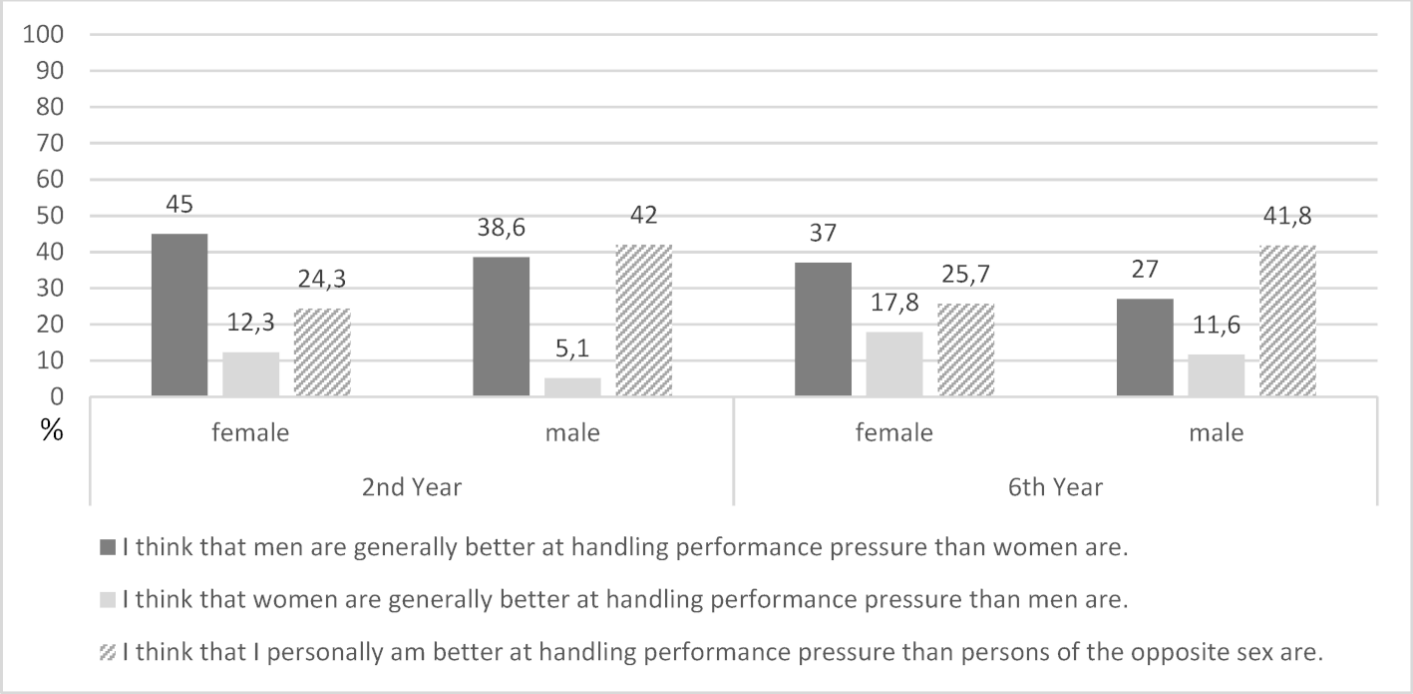
Agreement on gender-specific statements towards dealing with performance pressure. Agreement on gender-specific statements towards dealing with performance pressure of female and male students in %.

If students compare themselves with fellow students of the opposite sex, the result is clear: at both time points, significantly more male students than female students believe that they can cope better with performance pressure than students of the opposite sex. The described results on gender-stereotyped attitudes are shown in figure 2 (Fig. 2). 

## Discussion and conclusion

Students of both sexes experience performance pressure similarly, but it is more often stated that men can cope better with this pressure than women. In our survey, differences in problem perception were only found in the areas of competition and finance; female students in their 6th year experience these areas as problematic more often than their male colleagues. There were hardly any problems making social contacts. Problems with performance requirements are present mainly at the beginning of the studies, to a similar extent for men and women. Female students are particularly dissatisfied with their work-life balance in the 2^nd^ year group. Female and male students feel increasingly disadvantaged because of gender at the end of their studies. Fisher and Yao [18] examined financial risk tolerance in a study and found that women have less tolerance regarding financial risks and income insecurity. In our study, contrary to the results of Erschens et al. [15], there were few problems in the area of social contacts. This may be because classes take place in small groups from the beginning of studies and offer students the opportunity to work closely together and find social contacts or friendships. In both groups, men are considered more competent in dealing with performance pressure, even though female students perform at the same level in objective assessments as their male colleagues and there are hardly any differences in the problems male and female students experience. Babaria et al. report that women experience less confidence in their abilities and more anxiety about their performance [19]. In our study, about half of the surveyed students denied that there were differences between the two sexes in dealing with performance pressure, but among the other half, the opinion that men were superior to women in this respect was clearly predominant. This applied equally to male and female students and to students at the beginning and end of their studies. On the other hand, the statement that women are generally better at dealing with performance pressure than men, was very rarely accepted by both sexes. The female students also rated their personal ability to deal with performance pressure as rather low compared to men. Male students agree with this statement much more often. According to Kamas and Preston [20], a lack of confidence in one’s own ability to perform is often the reason why women avoid entering competitive situations. This is disadvantageous for the professional development of women. Abele [3] emphasizes that the professional expectation of self-efficacy can predict success. The “stereotype threat” phenomenon [7], [8], which is known from various areas, states that in areas in which one believes that other groups have better abilities, one’s own performance can be negatively impacted. The additional pressure resulting from this negative self-assessment could be a cause of the tendency towards low inner peace and balance, lower professional ambition and higher resignation, found by Aster-Schenck et al. [21], and at the same time play a role in the repeatedly documented higher psychological morbidity [22], [23], [24] of female students. Many of the male students also have problems with performance requirements, but this does not seem to affect their subjective feeling of being able to cope well with the demands of their studies. Haidinger et al [6] also identify “certainty of success”, i.e. self-confidence in approaching a task to be mastered as an important factor for actual success. Studying medicine is possibly still perceived as a male domain [9], in which male students move with greater confidence [6]. The feeling of men, that they are coping well with the demands of study fits in well in this concept. Miksch et al. [25] found that women perceive all examined aspects of work-life balance more relevant; they conclude that women are already more concerned with the double burden and compatibility of work and family during their studies. It is also striking that although competition among students at the beginning of their studies is hardly a problem, female students in particular experience competition more often as a problem at the end of their studies. The lack of traditional “rules of the game” for women in competitive situations could make it more difficult to deal with them. In addition, the tendency of women to want to avoid competitive situations [26] can play a role in the negative perception of competition. In the 6th year group, both sexes stated that they were significantly more frequently discriminated because of their sex – women even more frequently than men. Larsson [27] reports that gender-specific discrimination occurs above all in clinical internships, with an increased number of female students being affected, but not exclusively. This could also be the reason for the observed increase of competitive sensations and experienced discrimination in our 6th year group after the clinical internship. There is a trend towards women feeling more able to cope with the demands of their studies at the end of their training, and being more satisfied with their work-life balance. A more neutral attitude towards gender differences in dealing with performance pressure can also be observed among older students. This may be due to years of observation that women and men are equally successful in their studies. Abele [3] also notes feedback effects of success on self-efficacy expectations. Higher education policy measures, such as creating more female role models in visible positions, are, for example, an opportunity to promote women. The results of this study suggest that there is still a great need to address the importance and influence of gender roles and stereotypes in the curriculum and to encourage students to reflect their own attitudes.

## Limitations

The 6^th^ year group was significantly smaller than the group in the 2^nd^ year. Comparability is therefore limited. A more detailed analysis of the topics discussed here and a longitudinal study would be desirable for the future. As with all studies with voluntary participation, a selection bias cannot be ruled out. The absence of the statement “I think that women and men are equally good at dealing with performance pressure” may be regarded as a shortcoming of this study. However, it was possible to reject both gender-related statements and thus express a neutral attitude. This approach was chosen to minimize the effect of social desirability. Standardized questionnaires, which collect similar or related constructs, were deliberately omitted in order to directly collect attitudes to gender stereotypical statements. An attempt was made to counteract the disadvantages of self-developed questionnaires, such as lack of information on reliability or validity, by directly inquiring attitudes.

## Competing interests

The authors declare that they have no competing interests. 

## Figures and Tables

**Table 1:**
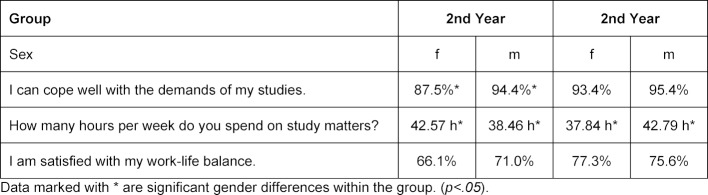
Agreement of female and male students with statements regarding study demands and working hours per week.
